# Ventral premotor cortex encodes task relevant features during eye and head movements

**DOI:** 10.1038/s41598-022-26479-2

**Published:** 2022-12-21

**Authors:** Ivan Smalianchuk, Neeraj J. Gandhi

**Affiliations:** 1 Department of Bioengineering, University of Pittsburgh, Pittsburgh, PA USA; 2 Department of Neuroscience, University of Pittsburgh, Pittsburgh, PA USA; 3 Center for Neural Basis of Cognition, University of Pittsburgh, Pittsburgh, PA USA

**Keywords:** Neuroscience, Cognitive neuroscience, Motor control, Oculomotor system

## Abstract

Visual exploration of the environment is achieved through gaze shifts or coordinated movements of the eyes and the head. The kinematics and contributions of each component can be decoupled to fit the context of the required behavior, such as redirecting the visual axis without moving the head or rotating the head without changing the line of sight. A neural controller of these effectors, therefore, must show code relating to multiple muscle groups, and it must also differentiate its code based on context. In this study we tested whether the ventral premotor cortex (PMv) in monkey exhibits a population code relating to various features of eye and head movements. We constructed three different behavioral tasks or contexts, each with four variables to explore whether PMv modulates its activity in accordance with these factors. We found that task related population code in PMv differentiates between all task related features and conclude that PMv carries information about task relevant features during eye and head movements. Furthermore, this code represents both lower-level (effector and movement direction) and higher-level (context) information.

## Introduction

Redirection of the visual axis to an object of interest causes its image to project on the fovea, the part of retina with greatest photoreceptor density, and permits visual inspection with high spatial acuity. Such changes in gaze are produced typically as a coordinated movement of the eyes-in-orbits and head-on-body. Previous reports on head and eye movements focus largely on the lawful relationship between the two effectors and less on features that allow them to be decoupled^1–6^. For example, we can keep eye contact and nod our head in agreement when talking to someone; or conversely, we can keep our head still and move just our visual axis to scan our environment. Additionally, we can perform the two movements in a sequence: nod first and then redirect the eyes within the orbits, or create a saccadic eye movement first and then nod. In both cases the individual eye and head movements are comparable, and yet the context for a movement of each is different. Even in conditions where we are not explicitly forcing a dissociation of the two effectors consciously, the interactions between eye and head movements can change depending on context^6–11^.

Investigations of the neural control of coordinated eye-head movements, let alone head-only movements dissociated from an accompanying gaze shift, are rare. Such studies have primarily focused on traditional oculomotor areas such as the superior colliculus (SC), the frontal eye fields (FEF), and supplementary eye fields (SEF)^5,12–20^. However, the general features of the movement, as well as a presence of a skeletomotor effector (head) led us to consider the role of a non-oculomotor area, the ventral premotor cortex (PMv), in the governance of head and eye movements. Stimulation of PMv produces eye and head movements that are often integrated with more complex and seemingly socially meaningful (e.g., defensive) movements of the hands and body^21–23^. These results were not presented in a traditional “eye movement” context as the focus was largely on skeleto-motor actions, and yet eye and head movements were produced. Additionally, neurophysiological work has reported modulation of PMv activity during eye and hand movements^24^. The results were presented from a hand–eye coordination viewpoint, rather than from an oculomotor perspective, but the parallels between hand–eye coordination and head-eye coordination reinforce our hypothesis that PMv encodes eye and head movement properties. More directly, Fujii et al. reported that saccades can be evoked by the electrical stimulation of the PMv^25^. Finally, a preliminary, trans-synaptic rabies virus injection study reported that PMv is only 3 or 4 synapses from eye and neck motoneurons^26^, suggesting possible control over those muscle groups. Given this set of results, we reasoned that PMv may also mediate movements of the eyes and the head, including instances when the two are decoupled.

The general consensus is that PMv does not exert direct control over movements. Its inactivation produces at most a mild change in a bias of movement direction and, in some cases, reduced EMG response in the fingers^27,28^. However, PMv does mediate higher-level properties for behaviors. For example, PMv has been shown to modulate its activity based on the sequence of motor actions in grasping^29^, reaching^30^, and even in linguistic domains^31^. Additionally, PMv neural activity differentiates between the contexts for movements. This has been primarily established in grasping domain, where several studies show that PMv modulates differently when a particular grasp is produced under different contexts, be it while performing the same grip to grasp different objects or producing the same grip but for different goals such as to eat a treat vs to just grasp^32^. Therefore, we hypothesize that PMv shows similar contextual, higher-level properties associated with eye and head movements.

Considering all the aforementioned information, we constructed an experimental design through which we assessed whether PMv participates in the generation of head and eye movements in a task-dependent manner. The design separated the gaze shift into individual head and eye movements performed in different order. We found significant correlations between single cell firing rates and task-related properties, which establishes the foundation for PMv role in eye and head movements. However, despite the statistical modulation, we could not identify any tuning properties in the activity of individual neurons. A population level analysis, in contrast and surprisingly, revealed a robust code for each individual task feature, which was additionally verified by a standard decoder. Collectively, these results show that PMv carries a population code for eye and head movement task features, such as the identity of the effector, the order of effector movement, and the hemifield to which the movement is directed.

## Methods

Data were obtained from one rhesus macaque (Macaca Mulatta). All procedures were approved by the Institutional Animal Care and Use Committee at the University of Pittsburgh and were in compliance with the U.S. Public Health Service policy on the humane care and use of laboratory animals. They are also in accordance with ARRIVE guidelines.

Surgical procedures were performed under aseptic conditions. A stainless-steel head post was affixed to the skull with bone cement and reinforced with titanium screws. A Teflon-coated coil wire was implanted around the sclera of one eye, and the leads of the wire were attached to a plug that was imbedded in the head post. Craniotomies were performed over the left and right PMv at coordinates estimated from MRI scans (stereotaxis coordinates: 15 mm anterior and 23 lateral to the right and left of the midline), and stainless-steel receptacles were placed over each opening. Behavioral and neural recording sessions were initiated following a recovery period after each surgery.

### Behavioral tasks

The animal sat in a custom primate chair approximately 100 cm from an LCD TV. The magnetic field induction method was used to sense eye position with the scleral search coil implanted around the eye. Head movements were tracked by placing another Teflon-coated coil on a custom-made pedestal on top of the head. To maintain consistency, we define eye and head movements in the external reference frame, which consequently equates an eye movement to a gaze shift. The animal received feedback about his head position via a live cursor on the screen, which is different from a head-mounted laser that we used in our previous works^33,34^. Reward, in the form of water or juice, was delivered through a tube that moved with his head^33^. Stimulus display and behavioral data acquisition at 1000 Hz were managed using custom software^35^.

**Figure 1:**
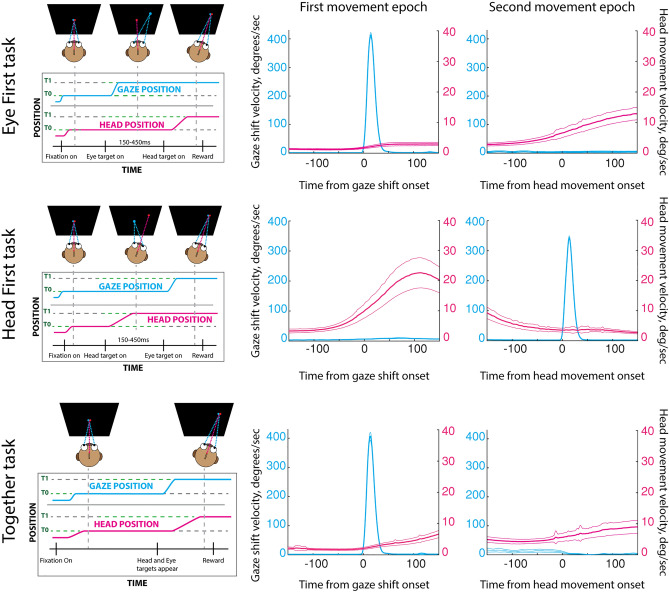
Task types performed by the animal. Top row: eye-first task. The animal was instructed to move the eye to a target location while keeping the head motionless. After a variable delay time, the animal had to orient the head to the same location as the gaze. Visual feedback of instantaneous head position was provided through a cursor on the screen. Middle row: head-first task. The animal first pointed its head to a target in the periphery by using the cursor feedback. After a variable delay the animal shifted its gaze to the same target. Bottom row: together task type. The animal aligned both head and gaze positions to a peripheral target. In this task the animal was not constrained in how he chose to complete the trial successfully. Feedback on head position was also provided for this condition. For each row, the middle and right panels show the average (over all sessions) gaze and head movement velocities during their movement epochs. Time is referenced in milliseconds. The thin lines are two standard errors from the mean.

Since we hypothesize that PMv encodes both motor- and context-related signals, we designed behavioral tasks to specifically differentiate these features. Special consideration was given to modifying the sequential order of eye-only gaze shifts and head-only movements. The tasks were designed in a way that kept characteristics of gaze shifts and head movements comparable across different trial types, but the context of the performed movement varied. Even in the most extreme cases (head velocity in eye-first and head-first tasks), the variance of behavior within each trial type was large enough yet the difference was low enough that much of the kinematics across the trial types overlapped (peak head velocities for eye-first: 20.2 deg/s, STD: 11.7 deg/s. head-first: 38.3 deg/s, STD: 27.1 deg/s); therefore we considered the kinematics across trial types similar. We reinforce this notion further in the manuscript by performing a subset of comparisons on trials that were matched by peak head velocity. This allowed us to assess whether PMv activity is associated with movement execution, task type, or a combination of both. In total, we constructed three different task types or contexts: “eye-first”, “head-first”, and “together” conditions (Fig. 1). These contexts were presented pseudo-randomly to approximately match the number of successful trials between all three types. All trials started with two fixation points 1° in diameter (red for head, white for gaze) appearing next to each other with no space in between. The animal positioned the head cursor within 3°-5° from the red target and directed gaze within 3° of the white target to initiate a trial.

In the “eye-first” case, the white point jumped from the center to one of four positions: (r = 10°, θ = 45°, 135°, 225°, 315°). This jump indicated to the animal to redirect its gaze to the new location while maintaining the head directed at the initial red point. After a variable delay (150–450 ms) the red point jumped to the same eccentric location as the white target (with a slight offset as to not overlap them). This indicated to the animal to align its head with the new location while maintaining gaze on the white point (Fig. 1, top). In the “head-first” condition the order of these events was reversed (Fig. 1, middle): the animal was instructed to first move its head, followed by an eye-only gaze shift. In the “together” condition, both points jumped to the eccentric position together and the animal was required to fixate and align the head cursor with the next location (Fig. 1, bottom). No constraints were placed on the relative timing of the two effectors.

### Neural data collection

**Figure 2:**
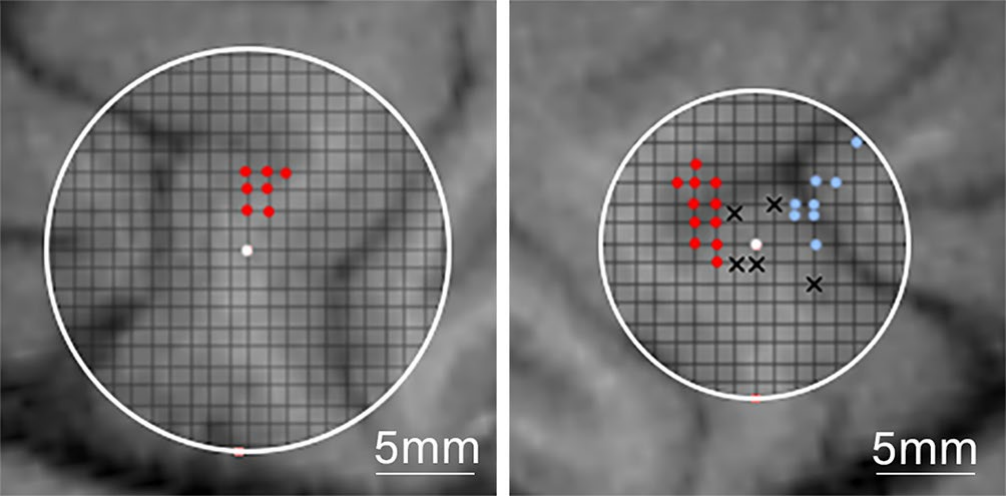
Recording and microstimulation sites. Left PMv (left) and right PMv (right) locations. The center point was denoted as the stereotaxic coordinates used in the craniotomy surgery (15 mm anterior, 23 mm lateral). The size of the perimeter is determined by the chamber used on each side. Inner diameter of the left chamber: 20 mm. Inner diameter of the right chamber: 15 mm. Red dots represent the sites of the cells used in this study. In blue are the sites where a clear eye movement was evoked with microstimulation. Black “x” marks represent sites where no behavior was evoked by electrical stimulation.

Electrophysiology experiments commenced once the animal performed the behavioral tasks consistently. In an initial set of experiments, a tungsten electrode was acutely lowered using a Narashige micromanipulator with an aid of a placement grid, which guided the penetrations through holes separated by 1 mm. To locate PMv within the chamber, we inserted the electrode in various grid locations, delivered electric pulses (20–100 µA, 50–300 ms, 200–400 Hz, biphasic pulses) and observed the evoked response, if any. We identified FEF as the region where microstimulation evoked a reliable saccade. We moved posterior one millimeter at a time until we no longer observed reliable FEF-like saccades, which indicated we were in PMv. In our case, we did not induce any saccades outside the FEF. Since PMv is a functionally diverse region, we aimed to only explore a specific area of interest (ROI) which corresponds to eye and head movements. We identified this area based on external works, notably that of Fujii et al.^25^, which placed our ROI approximately centered on the “spur” in the mediolateral axis, and ~ 1–4 mm posterior to the “spur”. This ROI also closely matches the polysensory zone described by Graziano and Gandhi^36^, which is associated with defensive movements which involve both head and eyes. Electrical microstimulation of this region in other studies evoked saccade-like eye movements with high reliability^24,25^, although we were not able to reliably elicit saccades in this region. We suspect the lack of microstimulation-induced behavior was a result in the differences in experimental design, notably due to the lack of a head restraint. Figure 2 shows the locations of the tracks that evoked saccades (blue) and those that did not evoke an identifiable movement (x shapes) superimposed over the MRI scans used for chamber placement coordinates.

After mapping the regions available within the chamber, we used Plexon™ 16 or 24 channel linear arrays with 200-micron intercontact distance to obtain neural recordings. We acutely lowered electrodes until we observed isolated neurons across a span of about 3 mm, or 15 channels. Well isolated neurons were seen on one to eleven separate channels, depending on the session. The locations of the recording sites are indicated in red in Fig. 2. Microstimulation did not evoke saccades at these sites. Although we did not systematically record the muscle activity of other effectors (e.g., arms, shoulders, torso, etc.), observation of the animal through a clear plate in the primate chair revealed no evoked movements. Data filtering, acquisition, and spike detection was handled by a Grapevine Scout system (Ripple Neuro). Raw signals were filtered with a 750 Hz high pass filter to remove spike-irrelevant signal, and spike detection was generally done using a 3RMS above the mean threshold. In some cases, a manual threshold was set to fine tune the detection of the spike. The channels with detected spikes were manually spike-sorted using MKsort (Ripple neuro); only one unit was isolated per channel as any additional units in this dataset were indistinguishable from noise.

### Data analysis

All data was analyzed in MATLAB using custom scripts. Eye movement data was smoothed with a 5 ms square kernel to remove any equipment noise. Saccade onset was detected when the eye velocity exceeded 35°/s and remained over the threshold for at least 8 consecutive milliseconds. Head movement onset proved to be more challenging, as the head rarely remained perfectly still, and head velocity was generally an order of magnitude slower than the eye. To detect head movement, we used an epoch starting 100 ms prior to head go-cue to ~ 1000 ms after. We smoothed the velocity profile with a 30 ms square kernel and normalized it to the range of 0–1. Head movement onset was defined when the normalized velocity reached a threshold of 0.3 and remained above the threshold for 15 consecutive milliseconds. A temporal shift of -5 ms was added to account for an apparent lag in detection. We then used visual inspection to remove any trials where automatic head or eye movement detection failed. An average of detected eye and head movement velocities is shown in Fig. 1 (middle and right columns).

A total of 86 isolated cells were recorded. Neural data was smoothed using a gaussian kernel with a σ = 10. To reduce the number of possible conditions we collapsed across directions by only considering whether the movement was ipsiversive or contraversive to the side of neuron being recorded. This reduction yielded a total of 12 different conditions: three task types $\times$ two directions (ipsi- and contraversive) $\times$ two effectors (eye or head). For example, one condition could be that the neural activity is modulated during eye-only saccades directed in the contraversive direction in the head-first task only.

Statistical significance of the neural modulation of each neuron was determined by a custom application of a paired t-test. We first grouped the trials into twelve separate categories. For each trial, we extracted a baseline period of 0–300 ms after the animal aligned both its gaze and head with the initial fixation targets. During baseline the animal was unaware of the context of the task. We then averaged each trial’s neural activity during the baseline period, giving us a $N\times 1$ vector ($A$) where $N$ is the number of trials, and each element is the mean baseline firing rate for that trial. For each trial’s movement epoch, we extracted the neural activity from − 80 ms to + 80 ms around movement onset resulting in an $N\times t$ matrix for each gaze and head movement (matrices $B$ and $C$, respectively), where $N$ has the same trial indices as vector $A$ and $t$ is time in millisecond increments. Then we conducted a paired t-test with $p$ threshold of 0.01 comparing each column of either matrix $B$ or $C$ to column vector $A$. We considered the cell to be significantly modulating for a specific category when the test determined significant difference in distributions for at least 20 consecutive timepoints. Using this method, we identified 74 out of 86 cells which significantly modulated their firing rate during at least one of the conditions.

For population level representation, we used dimensionality-reduction techniques on all 86 cells, thereby including the 12 neurons that did not show statistically significant modulation. Since these cells were recorded individually and across many sessions, we created a pseudo-population by arranging the data in such a way that the resulting structure can be treated as a simultaneously recorded set. For each neuron, we first categorized each trial into one of the three trial-types and the direction of the movements. Next, we took a snippet of activity centered on each effector movement onset (± 150 ms), one aligned on the gaze shift and the other aligned on head movement. We then created virtual trials by pulling either gaze-shift or head-movement snippets from 5 random trials from each cell’s dataset and averaging them to create representative activity for the pseudo- trial. The averaging step accomplished three crucial things. It diminished any outlier effects from noisy data, allowed us to increase the number of trials for sessions with low sample sizes, and reduced the possibility of repeated identical pseudo-trials. By repeating this process for each cell in the set, we attained one pseudo-dataset in which all cells can be treated as if they were recorded simultaneously. We created 500 virtual trials for each of the 12 conditions.

After constructing the pseudo-population, we used principal component analysis (PCA) to determine the first two principal components of the population activity which gave us insight into population activity patterns. PCA determines an axis of the multidimensional dataset which encompasses the highest amount of covariance across all neurons’ firing rates: the first principal component (PC). Given that high variance has an overall high ceiling for information capacity, one can hypothesize that the first PC encompasses the dimension with highest amount of information. This typically captures the most relevant patterns of the multidimensional signal and eliminates the patterns which carry less information. To establish the principal components, we concatenated all trials in the pseudo-population, giving us a $\left(301 \mathrm{ms} \times 500 \;trials \times 12 \;conditions\right)\times 86 \; neurons$ matrix. Applying PCA to this structure created a universal space for the entirety of the neural activity, onto which we could project activities of individual conditions to establish whether they reside in unique subspaces. We then plotted each trial (individual 301 ms snippets of the overall PCA matrix) corresponding to one of the 12 conditions aligned on movement onset to visualize any patterns of activity unique to that condition.

Although through this analysis it became evident that there are differences between conditions, we used PCA mainly as a visualization tool to gain insight into population activity patterns. To quantify the differences between high dimensional patterns corresponding to each condition, we used linear discriminant analysis (LDA) which finds the dimension of highest separation. We then determined whether there was significant pairwise difference between activities associated with each condition along that dimension. The linear discriminant model was developed using MATLAB’s Statistics and Machine Learning toolboxes. Since LDA is a pairwise discriminator, we created a separate model for each pair of conditions tested. As in PCA analysis, we concatenated the data across trials, but here we only used the subset of trials corresponding to the two selected conditions; thus, the resulting training matrix was: $\left(301 \mathrm{ms} \times [500 \; trials \; condition \; a+500 \; trials \; condition \; b\right]) \times 86 \; neurons$ with the corresponding label vector of $\left(301 \times [500 \; trials \; condition \; a+500 \; trials \; condition \; b\right])$ where each value indicates one of the 12 conditions for each ms sample. The resulting linear model maximizes the distance between category means while minimizing within-category variance, thus establishing pairwise separability. We then performed a t-test (p threshold < 0.001) on the distributions in the first latent factor to determine whether the means were significantly separable. To address a concern that the neural patterns are simply differentiating between kinematic differences across trial types, we performed some of these analyses on a subset of trials in which the distributions of peak head velocities between trail types were matched.

### Decoder

We wanted to determine whether the information in the PMv code was robust enough to assign a class to an unknown signal. Although we could determine this using LDA as a classifier, the linear process inherent in this method meant that we would have to make many pairwise comparisons to determine a class. And in the case where LDA could not reliably separate between classes, we would have to create some choice rule to maximize classification accuracy. Thus, we instead trained a naïve Bayes classifier to assign one of 12 categories to an input trial and assessed the resulting decoding accuracy. We chose this particular classifier because of its relative simplicity and expect similar results regardless of the classifier chosen.

To maximize the rigor with which to test the classifier, we created separate ‘training’ and ‘testing’ pseudo-populations. To remove any possibility of a trial appearing in both ‘training’ and ‘testing’ datasets, we only pulled random even numbered trials from individual sessions for the ‘training’ set and odd numbered trials for ‘testing’ set. This ensured that when obtaining the 5 random trials needed to create one pseudo-trial, none of these individual trials were duplicated between the two resulting sets. Since this cut our available data in half, we removed all sessions where we had less than 35 trials remaining for any condition, which reduced our population from 86 to 67 cells.

The naïve Bayes classifier was trained in a similar fashion as the LDA method, except all conditions were used simultaneously. The input matrix was the same structure as the one used for PCA, albeit with less dimensions: $\left(301 \mathrm{ms}\times 500 \;trials\times 12 \;conditions\right)\times 67 \;neurons$ and the label vector spanned all the resulting rows: $\left(301 \mathrm{ms}\times 500 \;trials\times 12 \;conditions\right)\times 1$. After the classifier was trained on a full dimensional ‘training’ set, we used individual pseudo-trials from the ‘testing’ set as an input to the resulting model. The structure of the input was: $301 \mathrm{ms}\times 67 \;neurons$ and the output was a 301-element vector classifying each millisecond sample of the trial as one of 12 conditions. The mode of the resulting vector was used to indicate the chosen category. We repeated this procedure for 250 pseudo-trials per category from the ‘testing’ set to gain an average classifier performance.

To establish the minimum number of cells required for accurate classification, we ranked cells by the magnitude of their projection onto the first PC of the population. We repeated our PCA procedure on the ‘training’ test to obtain this ranking, and then re-trained the decoder first on the data composed on just one highest ranking cell $\left(301 \mathrm{ms}\times N \;trials\right)\times 1 \;neuron$. Then we tested this classifier with the ‘testing’ data from the same neuron to obtain classification accuracy. Next, we trained a classifier on the top two best ranked neurons combined $\left(301 \mathrm{ms}\times N \;trials\right)\times 2 \;neurons$, then three best neurons, and so on, adding one neuron at a time until we reach full population.

### Cortical depth

To explore the features of this neural code even further, we asked whether some features varied as a function of depth within the cortex. After all, our linear probes spanned the entirety of the cortex cross-section and therefore we recorded from cells from different layers. It is important to note that this was not a part of the initial hypothesis and therefore the experimental design did not control for the depth of the neural recordings. Regardless, we could estimate the relative depth of each neuron from the channel it was recorded from. Since we attempted to span the entirety of the depth of the cortex when we inserted the electrodes, it is reasonable to assume that superficial channels resided in superficial layers, and deep channels resided in deep layers.

We assigned one of three depth markers to each neuron: ‘deep’, ‘middle’, and ‘superficial’ based on the channel they were recorded on. Most of the recording was done on deep channels, so to keep the number of cells in each group equal, the channel-to-label assignments were not equal. Channels 1–3 were considered ‘deep’, channels 4–9 were considered ‘middle’, and channels 10–16 were considered ‘superficial’. This gave us relatively the same number of neurons between ‘deep’ and ‘superficial’ neurons (24 for ‘deep’, 36 for ‘middle’, and 23 for ‘superficial’), thus allowing for a more direct comparison between the two groups.

For those sessions where 24 channel electrodes were used, only the first 16 channels were considered. Since the channel spacing was the same between 16 and 24 channel electrodes, no transformation of electrode distances was needed to directly compare the two. By and large, channel 1 depth was comparable between different electrodes as they were all lowered to a similar depth (~ 4000–5000 µm) as measured by a microdrive (Narashige). It is worth reiterating here that the depth was not controlled, and any variation in scar tissue at the insertion site and cortex deformation would make the depth measurements unreliable.

The primary question we addressed is whether the task relevant information is only present at a certain depth in the cortex. To establish this, we repeated the LDA analysis on the depth relevant subgroups of neurons. We focused on any differences in this separability as a function of depth.

## Results

Extracellular spiking activities of PMv neurons were recorded with a multicontact, laminar probe as a trained Rhesus monkey performed three, randomly interleaved tasks (Fig. 1). The ‘eye-first’ task required an eye-only saccade to a target followed later by a head-only movement to that location. The ‘head-first’ task required a head-only movement to an eccentric target followed by an eye-only saccade to that location. The ‘together’ task required a combined eye-head movement to a stimulus in the visual periphery. To establish the role of PMv in coordinated gaze shifts and head movements we analyzed 86 isolated neurons in the context of 12 separate categories: 3 tasks × 2 effectors × 2 movement directions (ipsiversive or contraversive). We report here analyses on activities of single neurons as well as on pseudo-populations constructed from these cells. Statistical analysis of individual neurons showed significant, although qualitatively unimpressive and uninterpretable, modulation during movement epochs, yet examination of these activities as a population, through PCA and LDA methods, showed a clear separation of the neural code by category. The robustness of this code was then verified by stressing the parameters of a Naïve-Bayes classifier.

### Single neuron analysis

**Figure 3:**
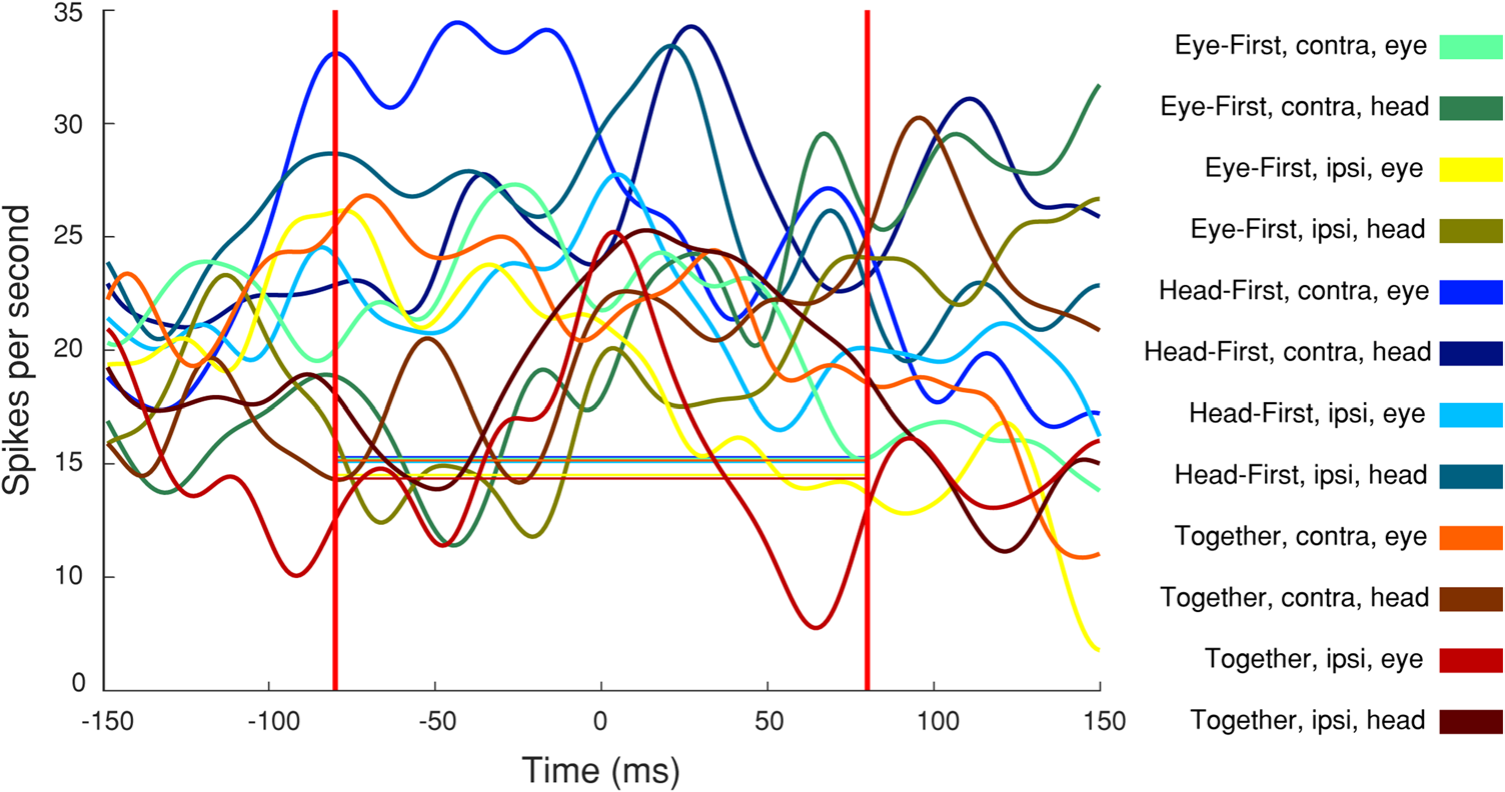
Timecourse of neural activity for an example PMv neuron. Trial-averaged firing rates for each of the 12 conditions are plotted as a function of time. The horizontal lines illustrate the average baseline activity for each condition, against which significance was determined in the epoch between the two vertical lines. Many baseline traces overlap, thus not all are detectable in the figure. No clear tuning across the 12 categories is identifiable in the firing rates of the neuron. The category associated with each color is shown on the right. The three pieces of information indicate trial-type, direction of movement being plotted, and the movement whose onset occurs at time 0. For example, “Eye-First, contra, head” category associated with the dark green color refers to the activity associated around the onset of a contraversive head movement in the eye-first task. This color-category designation is followed in subsequent figures.

**Figure 4:**
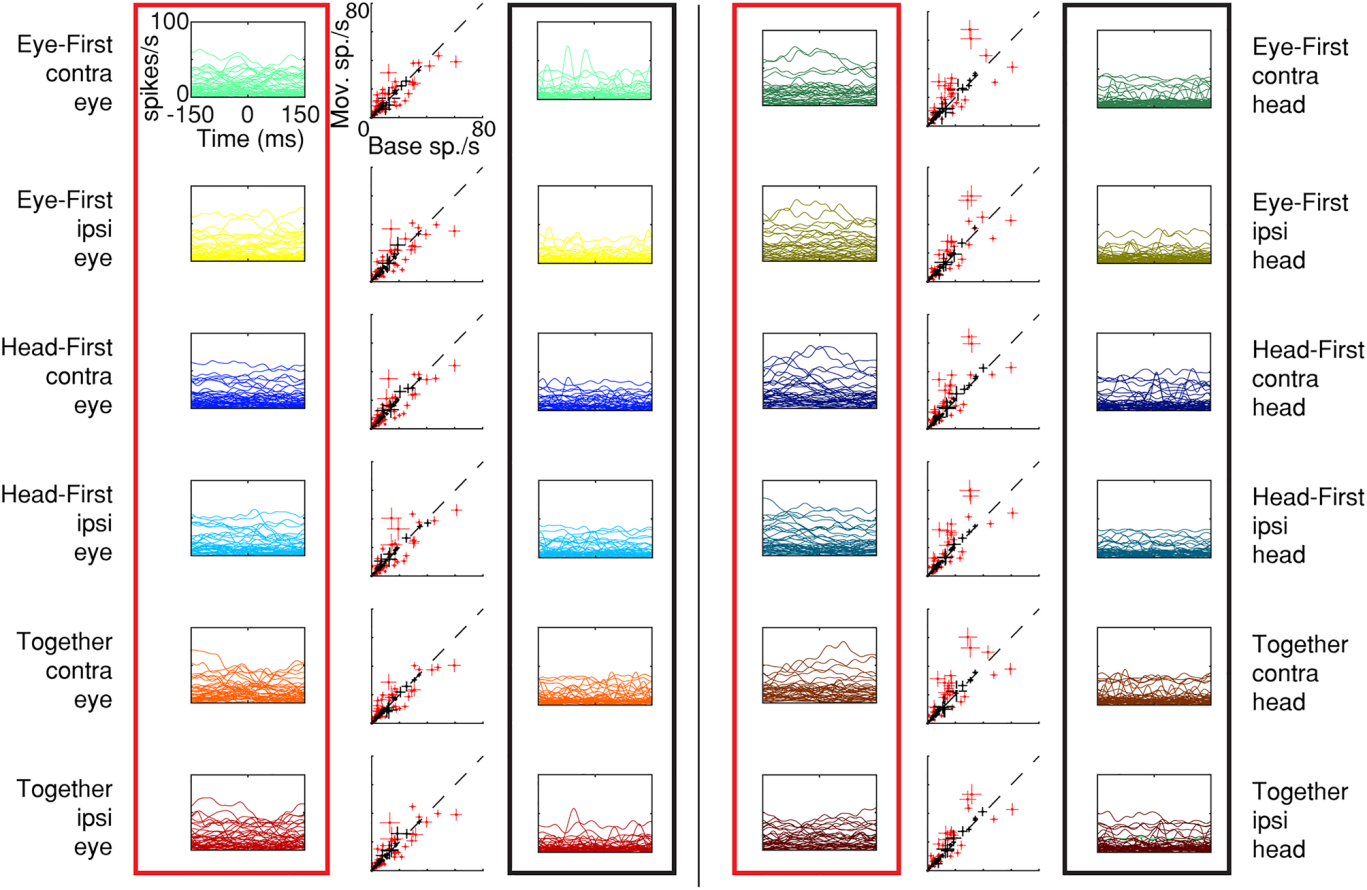
Distribution of cell responses during the movement epoch of each condition, grouped by statistical significance. Each scatter plot compares the trial-average activity during the movement epoch to the baseline period. Each point represents the mean and 95% confidence interval of one neuron. Red dots identify neurons with statistically significant modulation relative to baseline period, while black points denote cells that were not statistically modulated. The twelve scatter plots represent the 12 experimental categories, as denoted by text. The panels enclosed in the red boxes show temporal profiles of trial-averaged activity aligned on movement onset for the statistically modulated subset of neurons in the corresponding scatter plot. The panels within the black boxes show analogous data for the neurons that did not exhibit statistical significance.

We first compared each cell’s firing rate during the movement epoch (− 80 ms to + 80 ms from movement onset) to that of a baseline period (see methods). If 20 or more consecutive samples in the movement epoch were outside the baseline distribution (paired t-test, threshold p < 0.01), the cell was considered to have significant modulation for that condition. An example neuron’s spike density waveforms are shown in Fig. 3. In this case, the cell increased its firing rate above baseline for all categories, and no noticeable tuning for any one condition is evident. When examining average firing rates over time for all significantly modulated cells we notice the lack of features common to traditional motor areas; the cells neither exhibit burst-like properties, nor visible suppression signatures, nor any event-locked modulation (Fig. 4, left column). Qualitatively, the responses are flat throughout the movement epoch. To further illustrate this lack of apparent modulation, we contrast these cells with those that did not show significant modulation for a given category (Fig. 4, right column). One is hard pressed to identify concrete differences between the cells that were significantly active for each category and those that were not. In short, when examining individual cells, we see no obvious or characterizable relationship between the cells’ firing rates and the animal’s behavior.

**Figure 5:**
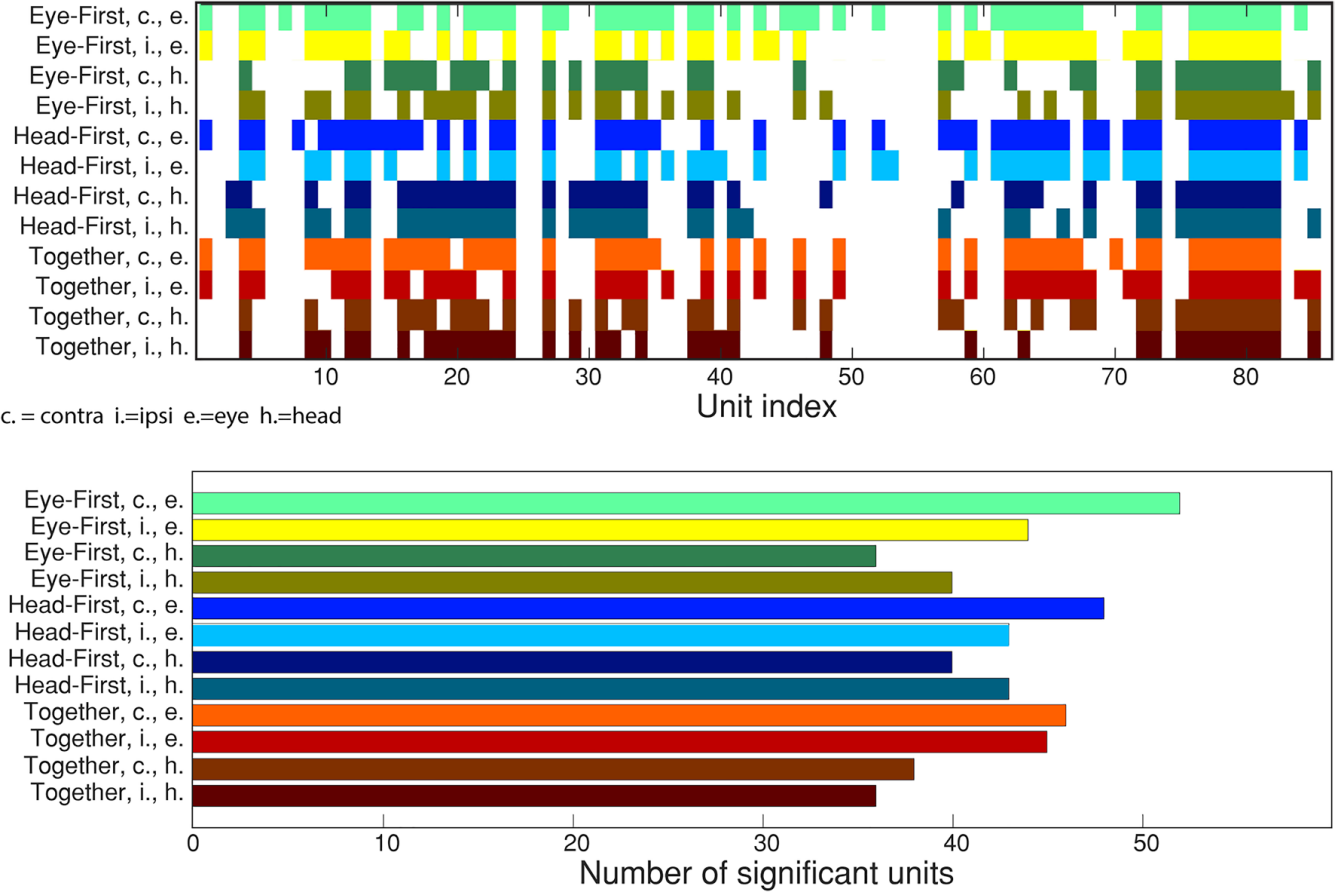
Heterogeneity of statistically significant modulation of PMv neurons by task conditions. Top: For each PMv neuron included in this study, we identify the conditions that produced statistically significant modulation relative to baseline as shaded pixels. Bottom: A horizontal histogram to denote the number of neurons with statistically significant modulation from baseline for each of the 12 categories.

We cannot discount statistical results based simply on visual inspection. To gain insight into the validity of the t-test results we plotted the significant activity compared to baseline activity for single cells (Fig. 4, middle column, red). We contrasted those results with cells in which the activity was not different from baseline in the same figure (Fig. 4, middle column, black). We can see here that all significant firing rate averages and their 95% confidence intervals lay far away from the unity line, thus signifying that this analysis was not overly contaminated with type 1 errors. Overall, we found that 74 out of 86 isolated cells showed a significant firing rate modulation from baseline during the movement epoch for at least one category (modified paired t-test, p < 0.01) (Fig. 5). This indicates that although visual examination of the data does not show any obvious or reliable patterns, the cells do in fact carry some information during the movement epochs. This information, however, is not immediately characterizable into a useable pattern.

### Pseudo population analysis

To gain a deeper understanding of the role PMv has in context-guided eye and head movements, we examined whether a clearer picture could be obtained if we considered the individually recorded neurons as a combined single population. We did not record all the cells at the same time. Each day the electrode was inserted acutely and therefore sessions across days had separate neurons recorded at different times. Luckily, the homogeneity of the tasks allowed us to restructure the data to simulate simultaneous recordings. We then used this pseudo-population with dimensionality reduction techniques to test for presence of a task-related code.

**Figure 6:**
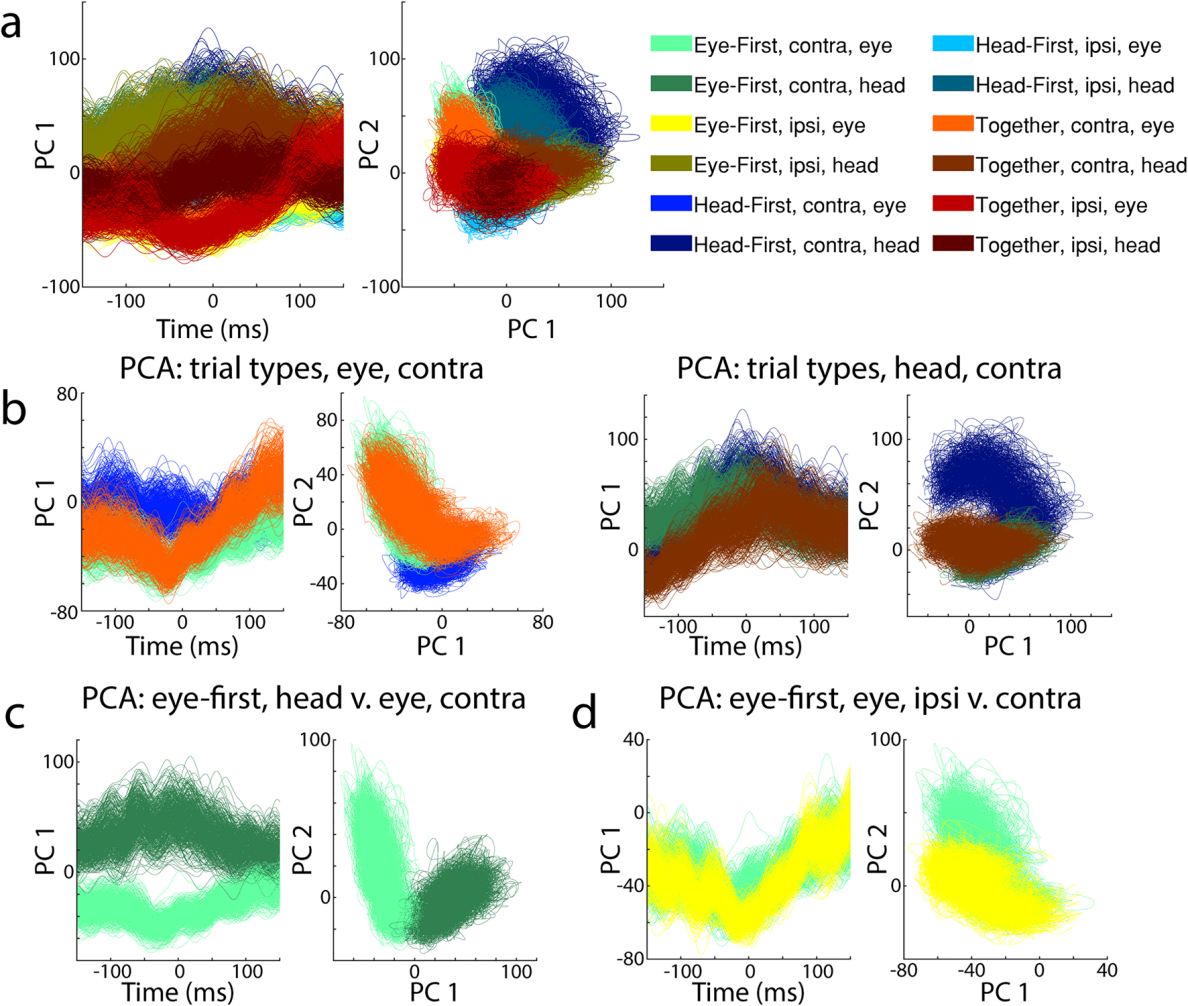
Representation of PMv pseudo-population activity using principal component analysis. (**A**) First principal components for all 12 categories are plotted as a function of time (left) or against the second principal components as a phase plane plot (right). (**B**) PCA results for the three trial types for data aligned on the onset of contraversive eye movements (left panels) and contraversive head movements (right panels). Color designation follows the convention established in previous figures: Eye-first (green), Head-First (blue), and together (orange) tasks. The bright colors represent PCs during the eye movement epoch, while the dark colors represent the head movement epoch. (**C**) PCA results differentiating patterns between the eye and head movement epochs. (**D**) PCA results differentiating between ipsiversive and contraversive eye movements.

We constructed a dataset which mimics a simultaneous 86 channel recording (see methods for details). For visualization purposes, we reduced the dimensionality of the data from 86 to top 2 dimensions using PCA. Figure 6 plots the first dimension as a function of time to illustrate any temporal patterns that may be present. We also plot PC1 against PC2 as a phase plane plot to illustrate any patterns in a multidimensional space. The legend (right) indicates the colors associated with each of the 12 conditions. As a guide, we used lighter colors to denote conditions associated with the eye movement epoch and darker colors for the head movement epoch. Unlike single neuron analysis for which the pattern of activity was impossible to characterize, the population activity clearly exhibits an unique trajectory for each category (Fig. 6A). When matching for all factors except trial type (Fig. 6B), we see that during each movement epoch, the activity traverses a different path during “head-first” task (blue traces) as compared to “eye-first” task (green traces). Additionally, the paths for “eye-first” and “together” task types are similar (compare light green vs. orange traces in the left panel of Fig. 6B). This could be because in natural gaze shifts like the ones we mimicked with the “together” task, the eye tends to move before the head and therefore resembles the “eye-first” task. Despite their similarities, we are still able to differentiate between the two trajectories. We observe these separable neural trajectories when we examine head movement epochs as well (Fig. 6B, right). The most pronounced difference, however, can be seen when we isolate the trajectories associated with head movement (Fig. 6C, dark green) and gaze shifts (light green), suggesting that representations for head movements and gaze shifts are exceptionally separatable in PMv population code.

For the sake of completeness, we examined whether the last remaining task-related factor (ipsi- vs. contraversive movement) is also encoded in PMv. Figure 6D shows that neural trajectories between contra- (green) and ipsiversive (yellow) movements appear to be most similar. However, the similarity between the trajectories is not surprising, as PMv has a large proportion of ipsilateral projections^37,38^ which could imply that a population code would not show a strong laterality preference. Regardless, the trajectories still show some differentiation (Fig. 6D).

**Figure 7:**
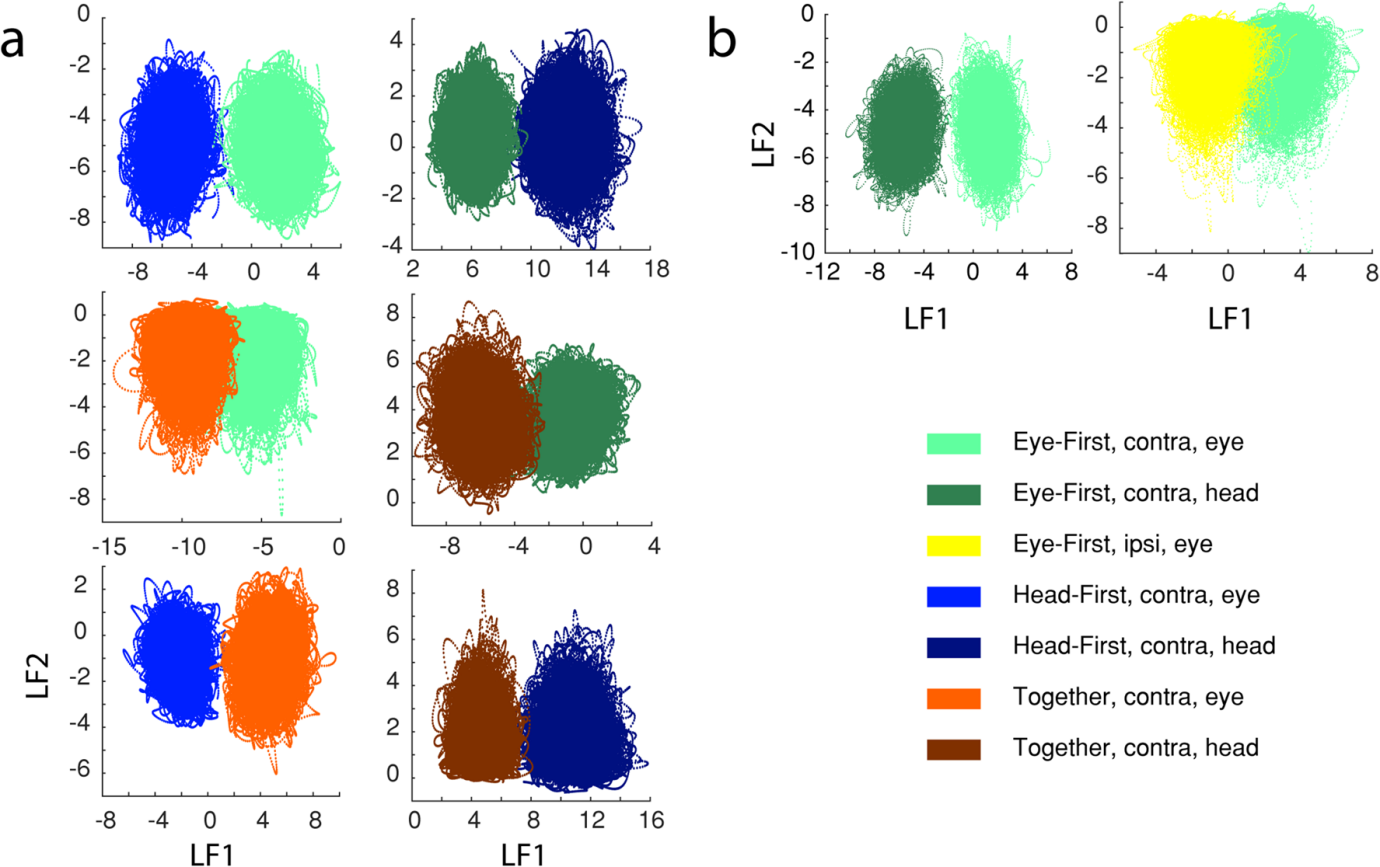
Representation of PMv pseudo-population activity using linear discriminant analysis. Each panel shows a phase plane plot of latent factor 1 (LF1) against latent factor 2 (LF2), with time implicitly represented in the trajectories. Each panel provides a pairwise comparison of two selected categories. (**A**) Comparisons of task-type conditions for contraversive eye movements (left column) and contraversive head movements (right column). LDA results show a difference in neural activity between head-first (blue), eye-first (green), and together (orange) trial types for eye (left column) and head (right column) movement epochs. The pairings and colors correspond to those found in Fig. 6B. (**B**) LDA results indicating separability of eye vs. head movement epochs (left) and ipsi- vs. contra-movements (right). The pairings and colors correspond to those shown in Fig. 6C,D.

Next, we used LDA to quantify the separability of the neural code during each condition. We found the dimension of maximum separability between pairwise-matched conditions and determined whether each condition’s neural activity was significantly different from the other using a standard t-test. Each pairing of conditions was significantly separable (p < 0.01). Figure 7 contains LDA results for the same conditions as we examined in Fig. 6B–D. In essence, here we show that PCA-visualizations of different neural trajectories traverse measurably separable neural subspaces. Since LDA performs best on pairwise comparisons, in Fig. 7A we compare differences in trial-types (eye-first, head-first, and together) in a pairwise fashion. Notice that even the neural representations of the conditions that seemed very similar in PCA results, namely the activity associated with eye-first and together trial-types, can be reliably separated using LDA (Fig. 7A, middle row). We repeated this analysis with neural activity representing head movements and gaze shifts, and, separately, ipsi- and contraversive movements (Fig. 7B). All pairwise matches showed significant separability. Note that even though we show a representative sample of these matchups, we performed LDA on all 24 relevant pairings and all were significantly separable (e.g., for the sake of clarity we do not show the results for ipsi- vs. contraversive movements for together, or head-first trial types in Fig. 7, but we have performed the calculations and found separability). We also analyzed LDA separability between eye-first and head-first trial types using a subset of trials with comparable movement parameters. When using trials from each condition in a way which resulted in equal distributions of peak head velocity across conditions, we still see a significant separation between trial types (with 86% accuracy). This is impressive, considering that this analysis reduced the number of useable trials by approximately half.

Our multi-dimensional analyses demonstrate that even though the single-cell analysis showed no clear pattern, there is a definite population code that becomes apparent when we visualize high dimensional data in lower dimensions. PMv neurons carry a task-relevant code for eye and head movements when considered as a population.

### Classification

**Figure 8:**
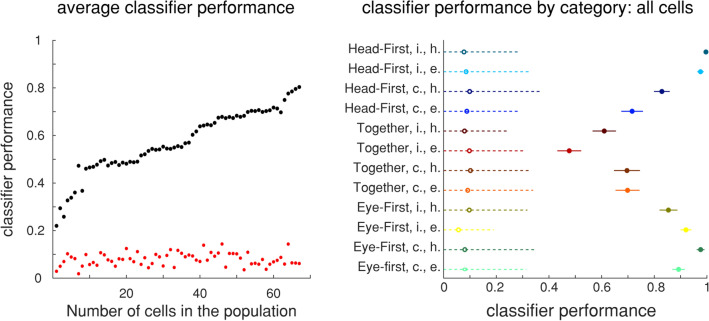
Naïve bayes decoder performance. Left: Black points indicate average decoder performance as a function of number of neurons used in the training population. Red points denote analogous information except when the training labels were randomized. Right: Mean decoder performance with two standard deviations span at full population separated by category. Open circles are performance from a decoder trained on shuffled data.

Although LDA shows a clear pairwise separation between different task features, it does not accurately reflect the way these neural signals are used in the brain. It is unlikely that a brain utilizes a pairwise decoder which compares between each pair of conditions individually. Therefore, we wanted to establish that PMv code is robust enough to categorize the activity into one of 12 categories at once. To accomplish this, we trained a relatively simple Naïve Bayes classifier (see “Methods”). When using all 67 cells for decoding we achieved relatively good results with an average accuracy of ~ 80%. The decoding accuracy was near 100% for some conditions, while slightly above 50% for others, still well above chance (Fig. 8, bottom).

We decided to test the robustness of this code by performing the classification on systematically increasing number of neurons. After sorting the neurons based on their first component weight of the PCA projection, we created a classifier on just one “best” neuron and evaluated its performance. We then continued to add neurons and evaluate subsequent classifiers. We see that it takes 45 neurons to achieve an average classifier performance of ~ 70%. In comparison, some neural classifiers from other cortical motor regions require as little as 15 neurons to achieve this accuracy^39^. This could suggest several possibilities: either the code lacks robustness, or the complexity of the task requires a large number of cells to encode it.

### Eye rotation in the orbits

One can apply a different interpretation of the results by taking a completely eye-centric approach. A difference in PMv neural activity in a contraversive eye movement and a contraversive head movement can be explained by the fact that in each case the eye rotates in a different direction in orbit: to the right in the former, and to the left in the latter. To account for this possibility, we compared the conditions where the eye rotation in orbit was in the same direction.

**Figure 9:**
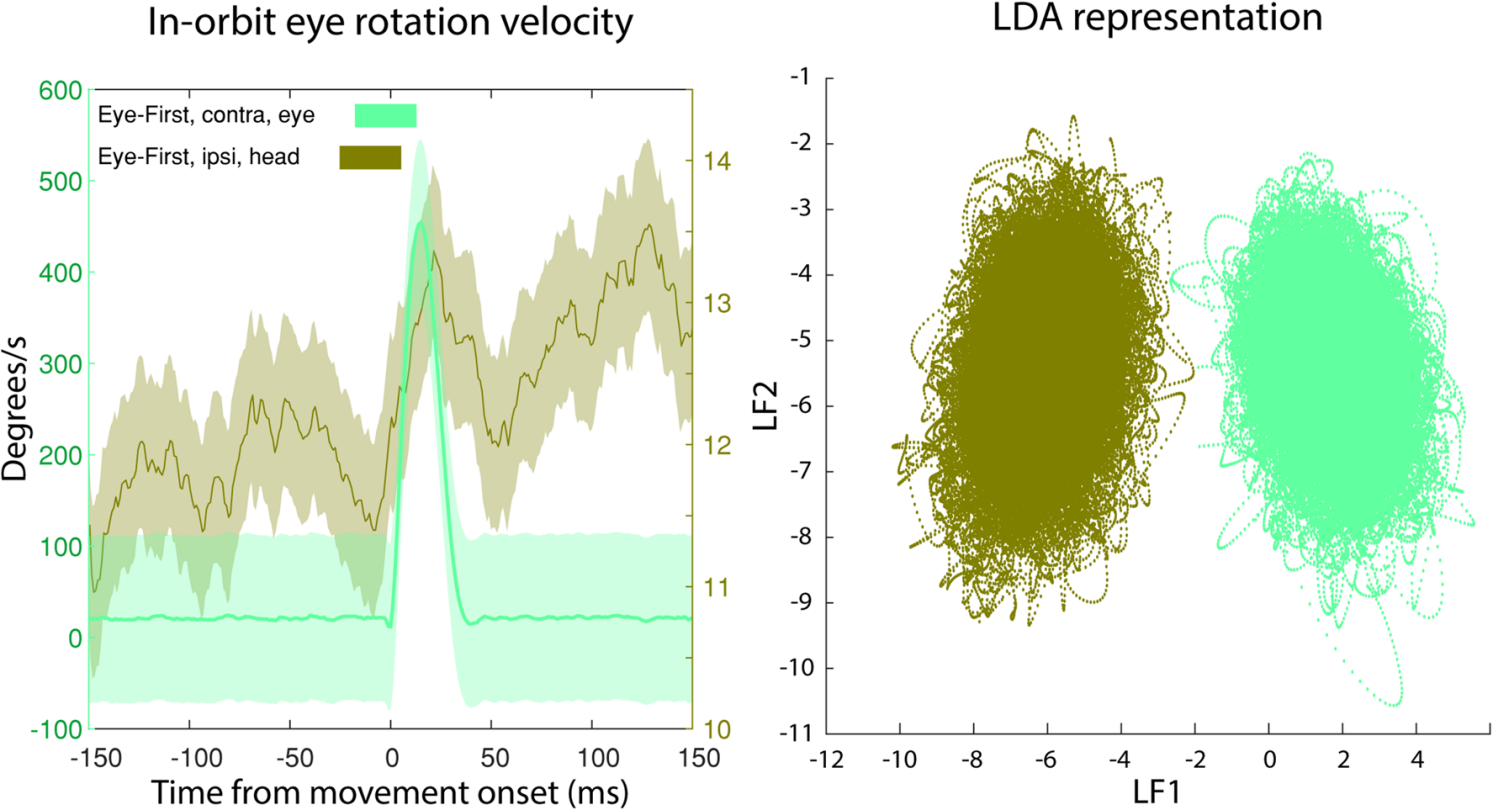
Comparison of the contraversive rotations of the eye in the orbits during separate conditions. Left: Eye-in-orbit velocity. Green line shows the eye velocity centered on eye movement onset for a contraversive movement on an eye-first condition. In this case the eye rotates in the head as a saccade in a contraversive fashion. Brown color represents the eye velocity as the head is making an ipsiversive movement. Thus, the eye is counter-rotating in the head in the contraversive direction, albite more slowly than in green condition. Shaded regions are 2 standard deviations around the mean. Note that different y-axis scales are used for the two traces. Right: LDA representations of PMv activity associated with the two conditions shown in panel A. The LDA results show clear separability in the population although the eye rotation in the head was in the same direction in both cases.

Figure 9 shows the case where we compared PMv activity in contraversive eye movements and ipsiversive head movements. In both cases the eye rotated in the ipsiversive direction in the orbit. We see that even when the rotation of the eye in orbit is in the same direction, the PMv activity is separable through LDA, indicating a difference in the population code for the two conditions.

### Cortical depth

**Figure 10:**
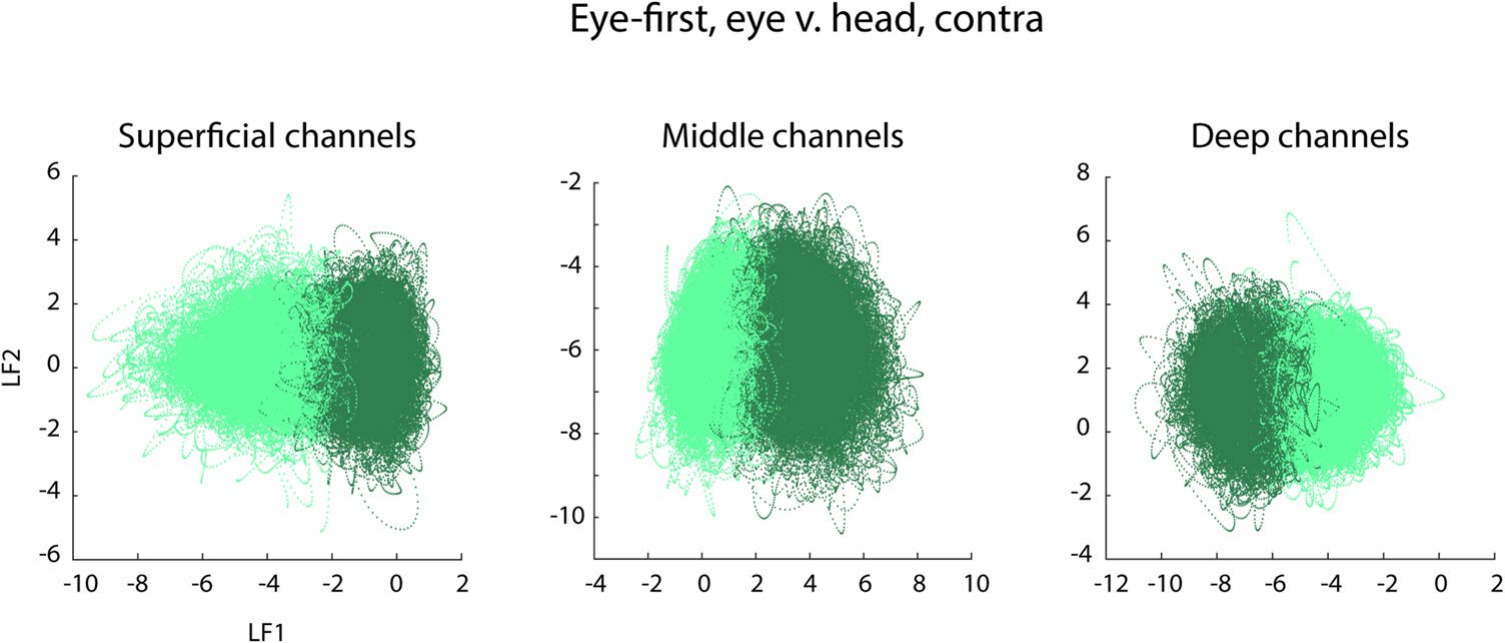
LDA analysis of eye vs head neural activity separated by relative cortical depth. Left subplot shows data from superficial layers, center subplot: middle layers, and right subplot: deep layers. We see that the quality of separation between the two parameters does not appreciably change based on the cortical depth.

When comparing the ability of PMv code to separate between task-relevant categories as a function of layers, we do not observe a quantifiable result. The separation between categories remains equal regardless of the depth of the recorded neuron; as an example, we show the separation between eye and head subspaces at different depths in Fig. 10. However, we cannot make a strong statement about this particular feature of PMv as our control for depth was poor.

## Discussion

The ventral premotor cortex exhibits a complex relationship with eye and head movement generation. During the exploration of the region via microstimulation, we observed no consistent effects on eye or head movements (see “Methods”). Single cell analysis uncovered modulation of neural activity during the movement epoch compared to a baseline period (Figs. 3, 4), but there was no obvious tuning by effector, task type, or direction of movement (Fig. 5). We also did not observe many of the familiar features present in traditional motor areas, such as movement-locked bursts^40–42^. In contrast, the pseudo-population analysis demonstrated the neural code can easily differentiate across the 12 different conditions we tested in our paradigm (Fig. 6). We also demonstrated that a simple decoder (Fig. 7) could access this code and determine the action (gaze shift or head movement), the direction of the movement (ipsiversive or contraversive), and the context under which this muscle group was recruited (eye-first, head-first, or together). Thus, we interpret our data to reveal that PMv does exhibit a clear code for changing gaze or head but through a framework that cannot be appreciated or recognized with single cell analysis.

Stimulation of the primary motor cortex evokes skeletomotor movements at short latencies. Finger movements can be produced with very low currents^43,44^. Eye and head movements can also be induced, typically as a coordinated gaze shift, with relatively low currents delivered to the superior colliculus and frontal eye fields. Regions that project to these primary motor structures, in contrast, require substantially stronger current parameters to generate a movement and, even then, the effect is inconsistent and heterogenous. One important reason for the departure from observing consistent stimulation-evoked movements is, we believe, the degradation of topography, which enables the region to gain additional functionality. Indeed, eye and/or head movements produced from the lateral intraparietal cortex and supplementary eye field require high currents, on the order of 5–10 times higher than SC and FEF, and the movements evoked seem more reflective of reference frame computations. Equally important, not every site in the region is equally effective at eliciting a stimulation-evoked movement. Stimulation of the dorsal premotor cortex (PMd) also evokes a variety of movements. Activation of skeletomotor musculature is possible with low currents, and a rough topography is indicated in some studies^23,45^. However, stimulation can also recruit the ipsilateral limb muscles and moreover have suppressive effects^46^, further emphasizing the increase in functional complexity.

A similar pattern has been described for movements evoked from PMv. The superior portion of PMv has large representations of forelimbs and shoulders, and inferior PMv contains a large orofacial representation^45^. However, orofacial and forelimb movements are also evoked from sites in superior PMv, indicating that the topography is coarse. The PMv is most closely associated with evoking complex and socially meaningful movements, which are observed only with long duration stimulation (500 ms) and large currents (> 100 μA). There also exist reports of ipsilateral head and goal-directed eye movements evoked by microstimulation of this region^21,24^ but the number of sites producing such effects is not big. We too reported previously similar effects and with low yield, and the results were comparable to previous reports^47^. Thus, we did not continue to systematically study the effects of microstimulation. We speculate that the coarse topography may be the key reason why stimulation of PMv, despite its close connectivity with primary skeletomotor and oculomotor areas, isn’t able to elicit movements reliably and homogenously.

Neural activity of PMv neurons exhibits ample heterogeneity as well. Given our experimental design, we were positioned to test for sensitivity of PMv neurons to innervation of multiple effectors, the impact of task type, and direction of movement. We first approached this examination through the lens of traditional oculomotor studies, analyzing firing rates of individual neurons aligned on stimulus and movement onsets. However, no such features were prevalent in the data. On visual inspections, cells did not seem to modulate the activity appreciably regardless of any experimental factor. There were many cases of statistical significance in firing rate modulation, but the magnitude of the effect was small and questionable for physiological significance. Cells in our database exhibited exceptionally distinct firing rate codes, and this heterogeneity obfuscated the mechanism by which these cells encode trial relevant features. Thus, we were not comfortable with attempting to extract a satisfactory description of PMv involvement in gaze shifts based on single unit analyses.

Previous studies have highlighted examples of PMv neurons that exhibit saccade related bursts during head-restrained eye movements produced either in isolation or in association with hand movements^24,25,48^. There are also many cases in which the activity modulation is modest at best and not burst-like^48^. We are not aware of previous studies that investigated PMv activity during head movements or head-unrestrained eye movements, but we do not believe the dearth of strong modulations in our dataset is due to the focus on a different effector system. We speculate that the complexity of our experimental paradigm may have altered the network properties of PMv neurons. Perhaps if the task was less complex, where the cells had to encode fewer parameters, then the firing rates may have exhibited different features. The notion is that if cells respond to multiple task parameters at once, their single-cell code might be obscured, and a population response may be needed to test our hypothesis. Hence, the logical next step in our data analysis was to examine the data as a unified pseudo-population. Indeed, a clear code was easily discernable. After a simple PCA dimensionality reduction, we could see the separation of neural trajectories as a function of task properties in the first two principal components (Fig. 6). This effect was even more obvious in an LDA projection of the data (Fig. 7).

The results of this study ascribe interesting properties to PMv neural code. It reveals that a weighted linear combination of PMv activity can be used to differentiate movement related activity for the eye (or gaze) from that of the head. Importantly, it does so with features—for example, an absence of bursts—that are different from traditional motor areas. Thus, it is likely that PMv activity is not controlling movement kinematics or dynamics, unlike the motor cortex^49–51^ and superior colliculus^52–56^. Our analysis also demonstrates that PMv can multiplex additional dimensions of information. Notably, the population response also encodes the context or task type and the directionality of the movement. These findings are consistent with insights gained through a machine-learning inspired approach to population level analysis in various cortical^51,57,58^ and subcortical^59^ areas. They also align with previous single-unit studies linking PMv involvement in determining context for actions, order of events, and decision making^27,31,60^: all roles which would not be out of place for PFC. This may corroborate the proposed functional connectivity between dorsolateral PFC, the PC, and M1^61^.

Although we show that PMv has task-relevant information spread across the neural population, we feel it is prudent to speculate further why we do not observe traditional firing rate properties on the single-cell level. As mentioned above, it is possible that the complexity of a particular task determines how a neuron encodes information about it. We know that some neural populations enter specific subspaces based on the nature of the task the subject is performing, as if to prime the population for an efficient encoding of the task that is to come^51,58^. It is possible that when a population is tasked to encode for a task with one or two factors, it can encode these factors in one or two features of firing rate. Here we can imagine that high firing rate could encode factor “A”, medium firing rate can encode factor “B”, and unchanged firing rate could mean the lack of either factor. Once the population is tasked with encoding 12 factors, a simple firing-rate based code might not be sufficient. In this case, the neural population can enter a mode in which it spreads the encoding of factors across many cells. And in a natural setting, PMv might need to monitor several dozens of features, which is practically impossible to do on a single-cell level. This presents a question, when we observe a specific encoding pattern in cortical areas, is that pattern task specific? Do encoding patterns which we record in a lab setting, where we distill behaviors to a few factors, transfer to natural environment, where behaviors depend on many conditions? The advances in recording technologies, wireless in-cage neural recordings for example^62^, should take us closer to answering these questions. In the meantime, we must be cognizant of the effects the task parameters have on the way the neural population encodes the behavior.

## Data Availability

The data that support the findings of this study are available from the corresponding author, N.J.G, upon reasonable request.
